# Decreased humoral immune response in the bronchi of rapid decliners with chronic obstructive pulmonary disease

**DOI:** 10.1186/s12931-022-02125-3

**Published:** 2022-08-03

**Authors:** Antonino Di Stefano, Francesca Dossena, Isabella Gnemmi, Silvestro Ennio D’Anna, Paola Brun, Bruno Balbi, Alessio Piraino, Antonio Spanevello, Francesco Nucera, Vitina Carriero, Francesca Bertolini, Mauro Maniscalco, Ian M. Adcock, Gaetano Caramori, Fabio L. M. Ricciardolo

**Affiliations:** 1 Divisione di Pneumologia e Laboratorio di Citoimmunopatologia dell’Apparato Cardio Respiratorio, Istituti Clinici Scientifici Maugeri, IRCCS, Respiratory Rehabilitation Unit of Veruno Institute, Via Per Revislate, 13, 28010 Veruno, NO Italy; 2 Divisione di Pneumologia, Istituti Clinici Scientifici Maugeri, SpA, Società Benefit, IRCCS, Telese, BN Italy; 3 Department of Molecular Medicine, Histology Unit, University of Padova, Padua, Italy; 4 Medical Affairs, Chiesi Italia SpA, Parma, Italy; 5 Divisione di Pneumologia, Istituti Clinici Scientifici Maugeri, SpA, Società Benefit, IRCCS, Tradate, VA Italy; 6 Pneumologia, Dipartimento di Scienze Biomediche, Odontoiatriche e delle Immagini Morfologiche e Funzionali (BIOMORF), Università di Messina, Messina, Italy; 7 Department of Clinical and Biological Sciences, Rare Lung Disease Unit and Severe Asthma Centre, San Luigi Gonzaga University Hospital, University of Turin, Orbassano, Turin, Italy; 8 National Heart and Lung Institute, Imperial College London, London, UK

**Keywords:** Airway inflammation, Sustainers, Functional FEV1 decline, pIgR, IgA, Plasma cells, CD20

## Abstract

**Background:**

Identification of COPD patients with a rapid decline in FEV1 is of particular interest for prognostic and therapeutic reasons.

**Objective:**

To determine the expression of markers of inflammation in COPD patients with rapid functional decline in comparison to slow or no decliners.

**Methods:**

In COPD patients monitored for at least 3 years (mean ± SD: 5.8 ± 3 years) for lung functional decline, the expression and localization of inflammatory markers was measured in bronchial biopsies of patients with no lung functional decline (FEV1% + 30 ± 43 ml/year, n = 21), slow (FEV1% ml/year, − 40 ± 19, n = 14) and rapid decline (FEV1% ml/year, − 112 ± 53, n = 15) using immunohistochemistry. ELISA test was used for polymeric immunoglobulin receptor (pIgR) quantitation “in vitro”.

**Results:**

The expression of secretory IgA was significantly reduced in bronchial epithelium (p = 0.011) and plasma cell numbers was significantly reduced in the bronchial lamina propria (p = 0.017) of rapid decliners compared to no decliners. Bronchial inflammatory cell infiltration, CD4, CD8, CD68, CD20, NK, neutrophils, eosinophils, mast cells, pIgR, was not changed in epithelium and lamina propria of rapid decliners compared to other groups. Plasma cells/mm^2^ correlated positively with scored total IgA in lamina propria of all patients. “In vitro” stimulation of 16HBE cells with LPS (10 μg/ml) and IL-8 (10 ng/ml) induced a significant increase while H_2_O_2_ (100 μM) significantly decreased pIgR epithelial expression.

**Conclusion:**

These data show an impaired humoral immune response in rapid decliners with COPD, marked by reduced epithelial secretory IgA and plasma cell numbers in the bronchial lamina propria. These findings may help in the prognostic stratification and treatment of COPD.

## Introduction

Chronic obstructive pulmonary disease (COPD) is characterized by a progressive airflow limitation that is not fully reversible [1]. FEV_1_, FEV1/FVC and the rate of annual decline in FEV_1_ are the most widely used outcome measures for clinical trials and observational studies of COPD [2]. At present, factors known to affect the annual decline in FEV_1_ are smoking status [3, 4], frequency of exacerbations [5], pharmacologic therapies [6, 7] and severity of emphysema [3, 8]. Fletcher et al. reported that some patients with COPD might have lung function improvements over time [9] and, more recently, one study showed that 15% of COPD patients improved their lung function over a 3-year period [10] even though this finding was not clearly linked to factors potentially influencing lung function decline [10]. Biomarkers could play an important role in relation to lung function decline in terms of predicting changes in FEV1 [10] and guiding the best therapeutic approach for each COPD phenotype based on the extent of lung function decline. Increased blood levels of IL-4 and MCP-1 have been reported in rapid decliners compared to slow decliners with COPD [11]. Another study found that blood levels of neutrophils, CRP and MMP9 proteins were higher in rapid decliners than non-decliners [12]. In urine samples, desmosine and isodesmosine levels were lower in rapid decliners vs. slow decliners [13]. To our knowledge, no data are available on the pathology of COPD phenotypes according to the rate of lung function decline.

Immunoglobulin A (IgA) is the predominant immunoglobulin in bronchial mucosa, and bronchial epithelium transports polymeric Ig within the mucosal lumen via the polymeric Ig receptor (pIgR) of epithelial origin [14]. In the luminal environment, secretory IgA nhibits adherence of microorganisms to epithelial cells by the so-called “immune exclusion” mechanism [14, 15]. Mucosal plasma cells produce mostly polymeric IgA, mainly dimeric, which is also the predominant type of IgA (~ 80%) in secretions. It associates, in turn, with the secretory component of epithelial origin to form secretory IgA [14, 15]. In eight severe COPD patients, immunostaining for secretory component showed it to be significantly decreased compared to controls (n = 5), and the reduced expression of secretory component in large airways correlated with neutrophil infiltration in submucosal glands [16]. More recently, Polosukhin et al. examined areas of bronchial mucosa covered by normal and altered pseudostratified epithelium in COPD patients and found that altered bronchial epithelium had a reduced pIgR expression, and secretory IgA deficiency was associated with increased inflammation [17]. Patients with COPD also had reduced secretory IgA in bronchoalveolar lavage [17]. Patients who died from COPD were found to have a lower number of IgA + plasma cells in the bronchial mucosa than COPD patients who died of other causes [18].

However, to our knowledge, there are no pathology data available from bronchial biopsies of COPD patients stratified according to lung function decline (rapid, slow, or no decline) as regards the humoral immune response and inflammation. We thus aimed to quantify and compare the expression of inflammatory cells and markers of humoral immune response in bronchial biopsies of COPD patients with rapid vs. slow vs. no functional decline (sustainers). We used immunohistochemistry and an “in vitro*”* bronchial epithelial cell model to examine the impact of inflammation on the release of pIgR.

## Methods

### Subjects

All COPD subjects were recruited from the Respiratory Medicine Unit of the “Istituti Clinici Scientifici Maugeri” (Veruno, Italy). Archival material was used in the present study. We obtained bronchial biopsies from 50 patients for this immunohistochemical study. Their characteristics are reported in Table 1. COPD and chronic bronchitis were defined according to international guidelines: i.e., COPD = presence of a post-bronchodilator forced expiratory volume in one second (FEV_1_)/forced vital capacity (FVC) ratio < 70%; chronic bronchitis = presence of cough and sputum production for at least 3 months in each of two consecutive years [goldcopd.org]. In COPD patients, the severity of the airflow obstruction was graded using the 2011 GOLD criteria (goldcopd.org). Since standardized cut-off values for lung function decline are not available, we adopted cut-off values similar to those reported in the literature [7, 8, 10, 11], even though arbitrarily chosen. We defined rapid decline as rate of FEV1 decline > 70 ml/year over a period of 3–15 years, slow decline as FEV1 decline > 20 ≤ 70 ml/year, and no decline (sustainers) as FEV1 decline ≤ 20 ml/year, over the same period 3–15 years.

**Table 1:** Characteristics of COPD patients used for bronchial biopsy analysis, according to lung function decline

Patients with COPD	No decline	Slow decline	Rapid decline
Number	21	14	15
Age	68 ± 8	67 ± 8	66 ± 7
M/F	19/2	11/3	15/0
Follow-up duration for functional decline evaluation (years)	6.1 ± 3	6.8 ± 4	4.4 ± 2
Pack-years (ex/current smoker)	47 ± 29 (11/10)	40 ± 33 (10/4)	65 ± 30^^^ (6/9)
Frequent exacerbators in the year before bronchoscopy (n/%)	6/28%	0/0%	3/20%
FEV_1_ pre-β_2_ (% predicted)	62 ± 19	55 ± 17	53 ± 19
FEV_1_ post-β_2_ (% predicted)	64 ± 15	56 ± 12	55 ± 15
FEV_1_/FVC % pre-β_2_	53 ± 10	52 ± 13	48 ± 12
FEV_1_/FVC % post-β_2_	54 ± 14	54 ± 8	49 ± 7
Functional decline FEV_1_% post- β_2_ (ml/year)	+ 30 ± 43	− 40 ± 19^*^	− 112 ± 53^&^
RV%	162 ± 34	171 ± 47	166 ± 35
DLCO%	73 ± 18	67 ± 21	52 ± 14^#^
DLCO/VA%	70 ± 15	61 ± 22	54 ± 9
CT-scored emphysema %	17 ± 22	20 ± 19	34 ± 25

Data are expressed as mean ± standard deviation. ^^^significantly different from slow decliners and non-decliners with COPD (p < 0.05); ^*^Significantly different from non-decliners with COPD (p < 0.0001); ^&^significantly different from slow decliners and non-decliners with COPD (p < 0.0001); ^#^Significantly different from non-decliners with COPD (p < 0.05); FEV_1_: forced expiratory volume in 1 s; FVC: forced vital capacity; RV: residual volume; DLCO: diffusing capacity for carbon monoxide; DLCO/VA: diffusing capacity for carbon monoxide/alveolar volume

All COPD patients were stable and none were treated with theophylline, antibiotics, antioxidants, mucolytics, and/or glucocorticoids in the month prior to bronchoscopy. The study conformed to the Declaration of Helsinki, and was approved by the Institutional Review Boards of Istituti Clinici Scientifici Maugeri (protocol p112).

### Lung function tests and volumes

Pulmonary function tests were performed as previously described [19] according to published guidelines [19, 20]. Pulmonary function tests included measurements of FEV_1_ and FEV_1_/FVC under baseline conditions in all the subjects examined (6200 Autobox Pulmonary Function Laboratory; A number of two to 7 pulmonary function tests were evaluated for each patient over the period of 3–15 years considered for evaluation of lung functional decline. Sensormedics Corp., Yorba Linda, CA). In order to assess the reversibility of airflow obstruction and post bronchodilator functional values, we repeated the FEV_1_ and FEV_1_/FVC% measurements in the groups of subjects with FEV_1_/FVC% < 70% pre-bronchodilator 20 min after the inhalation of 0.4 mg of salbutamol.

### Fiberoptic bronchoscopy, collection and processing of bronchial biopsies

Patients attended the bronchoscopy suite at 8.30 AM after having fasted from midnight and were pre-treated with atropine (0.6 mg IV) and midazolam (5–10 mg IV). Oxygen (3 l/min) was administered via nasal prongs throughout the procedure and oxygen saturation was monitored with a digital oximeter. Using local anesthesia with lidocaine (4%) to the upper airways and larynx, a fiberoptic bronchoscope (Olympus BF10 Key-Med, Southend, UK) was passed through the nasal passages into the trachea. Further lidocaine (2%) was sprayed into the lower airways, and four bronchial biopsy specimens were taken from segmental and subsegmental airways of the right lower and upper lobes using size 19 cupped forceps. Bronchial biopsies for immunohistochemistry were gently extracted from the forceps and processed for light microscopy as previously described [19]. At least two samples were embedded in Tissue Tek II OCT (Miles Scientific, Naperville, IL, USA), frozen within 15 min in isopentane pre-cooled in liquid nitrogen, and stored at − 80 °C. The best frozen sample was then oriented and 6 μm thick cryostat sections were cut for immunohistochemical light microscopy analysis and processed as described below.

### Immunohistochemistry on OCT-embedded bronchial biopsies

Sections from each sample were stained with antibodies specific for inflammatory cells and biomarkers of humoral immunity (Table 2). Briefly, after blocking non-specific binding sites with serum derived from the same animal species as the secondary antibody, the primary antibody was applied at optimal dilutions in TRIS-buffered saline (0.15 M saline containing 0.05 M TRIS-hydrochloric acid at pH 7.6) and incubated 1 h at room temperature in a humid chamber. Antibody binding was detected with secondary anti-mouse (Vector, BA 2000) or anti-rabbit (Vector, BA 1000) antibodies followed by ABC kit AP AK5000, Vectastain and fast-red substrate (red color) or ABC kit HRP Elite, PK6100, Vectastain and diaminobenzidine substrate (brown color). Nasal polyp sections were used as positive controls. For the negative control, normal mouse (sc-2025) or rabbit (sc-2027) non-specific immunoglobulins (Santa Cruz Biotechnology, Santa Cruz, CA, USA) were used at the same protein concentration as the primary antibody.

**Table 2:** Primary antibodies and immunohistochemical conditions used for identification of inflammatory cells and biomarkers of humoral immunity in bronchial biopsies

Antibody	Code	Species	Concentration
Neutrophilic elastase (neutrophils)	Dako, M752	Mouse	1:100
CD68 (macrophages)	Dako, M814	Mouse	1:300
ECP (eosinophils)	Pharmacia, EG2	Mouse	1:100
Mast cells (tryptase)	Invitrogen, MA538007	Rabbit	1:400
CD4	Santa Cruz, SC-19641	Mouse	1:40
CD8	Thermo Fischer, RM 9116	Rabbit	1:80
CD20	Dako, M755	Mouse	1:200
NK (natural killer)	Dako, M1014	Mouse	1:50
Plasma cells	Dako, M7077	Mouse	1:25
IgA	Gene Thex, GTX22411	Rabbit	1:200
IgA secretory	Abcam, AB17921	Mouse	1:50
pIgR	Invitrogen, PAS35340	Rabbit	1:20

### Scoring system for immunohistochemistry in the bronchial biopsies

Morphometric measurements were performed with a light microscope (Leitz Biomed, Leica Cambridge, UK) connected to a video recorder linked to a computerized image system (Quantimet 500 Image Processing and Analysis System, Software Qwin V0200B, Leica). Light-microscopic analysis was performed at a magnification of 630×.

The immunostaining for all the antigens studied was scored from 0 (absence of immunostaining) to 3 (extensive intense immunostaining) in the intact (columnar and basal epithelial cells) bronchial epithelium, as previously described [19]. The final result was expressed as the average of all scored fields performed in each biopsy. A mean ± SD of 0.700 ± 0.260 mm of epithelium was analyzed in COPD patients.

Immunostained cells in the bronchial lamina propria were also quantified 100 μm beneath the epithelial basement membrane in several non-overlapping high-power fields until the whole specimen was examined. The final result, expressed as the number of positive cells per square millimeter, was calculated as the average of all the cellular counts performed in each biopsy. Quantitation of the inflammatory cells and scoring of IgA, secretory IgA and pIgR were all performed (ADS) in a blinded fashion.

### Cell culture and treatments

We used the SV40 large T antigen-transformed 16HBE cell line which retains the differentiated morphology and function of normal human bronchial epithelial cells (NHBE) [19]. 16HBE cells were maintained in Dulbecco’s modified minimum essential medium (DMEM), supplemented with 10% v/v fetal bovine serum (FBS), 50 IU/ml penicillin, 50 µg/ml streptomycin, 1 × non-essential amino acids, 1 mM sodium pyruvate and 2 mM glutamine (37 °C, 5% CO_2_) (27). When cells were 70–80% confluent, the complete medium was replaced with DMEM with 1% FBS for starvation time (24 h). 16HBE cells were cultured for 0–24 h because of their lower resistance to starvation. Non-treated 16HBE cells were used as controls. All experiments were performed in triplicate, i.e., three independent experiments for each type of treatment [LPS, H_2_O_2_, IL-8 (CXCL8)] and each time exposure (4–12–24 h).

### ELISA tests in the supernatants and cell lysates of LPS, H_2_O_2_ and IL-8 treated and non-treated 16HBE cells

Human pIgR [Fine Biotech Co. ELISA (Cat. No. EH1460), detection range: 31.25–2000 pg/ml, lower detection limit: 18.75 pg/ml] protein quantification in the supernatants and cell lysates of LPS (SIGMA L9143, 10 µg/ml) H_2_O_2_ (SIGMA-ALDRICH 18304, 100 µM) and IL-8 (PreproTech CXCL8, 10 ng/ml) treated and non-treated 16HBE cells was performed as reported in the results section. ELISA kits were used according to the manufacturer’s instructions.

### Statistical analysis

Group data were expressed as mean ± SD (standard deviation) for functional data or median (range) or interquartile range (IQR) for morphologic data. Differences between groups were analyzed using analysis of variance (ANOVA) for functional data. ANOVA was followed by an unpaired t-test for comparison between groups. The Kruskal–Wallis test was applied to the morphologic data followed by a Mann–Whitney U-test for comparison between groups. In vitro data were analyzed by the Mann–Whitney U test. Correlation coefficients were calculated using the Spearman rank method. Probability values of p < 0.05 were considered significant. Data analysis was performed using the Stat View SE Graphics program (Abacus Concepts Inc., Berkeley, CA-USA.

## Results

### Clinical characteristics of subjects providing bronchial biopsies

We obtained and studied bronchial biopsies from 50 subjects with COPD: 21 with close to normal lung functional decline (i.e., non-decliners or sustainers), 14 slow decliners, and 15 rapid decliners (Table 1). Follow-up duration for functional decline evaluation was on average 6.1 ± 3, 6.8 ± 4 and 4.4 ± 2 years (ANOVA: p = 0.060) for non-, slow and rapid decliners, respectively (Table 1). Functional decline of FEV1% predicted post β2 was + 30 ± 43, − 40 ± 19 and − 112 ± 53 ml/year for non-, slow and rapid decliners, respectively (Kruskal–Wallis: p < 0.0001). Smoking habit, expressed as pack/years was higher in rapid decliners than in slow or non-decliners (Kruskal–Wallis: p = 0.045). DLCO% was slightly but significantly lower in rapid decliners than in non-decliners (Kruskal–Wallis: p = 0.046, Mann–Whitney: p = 0.019). When all patients were grouped together, the % emphysema scores correlated inversely and significantly with DLCO/VA% (R = − 0.635, p = 0.011). Table 3 shows the pharmacologic therapies prescribed in patients before and after bronchoscopy (at discharge). ICS is reported as beclomethasone dipropionate equivalent dose (μg/day). After discharge, therapy remained unvaried in the follow-up period in 17 patients with no functional decline (80.9%), 11 patients with slow functional decline (78.6%) and in all 15 patients with rapid functional decline (100%) (Table 3). ICS use at discharge was 1009 ± 539, 880 ± 268, and 1722 ± 754 μg/day BDP pMDI in non-decliners, slow, and rapid decliners, respectively (Kruskal–Wallis: p = 0.060, Mann–Whitney, non-decliners vs. rapid decliners: p = 0.036).

**Table 3:** Therapy prescribed in COPD patients before bronchoscopy and at discharge after bronchoscopy

	No decline (n. patients)	Slow decline (n. patients)	Rapid decline (n. patients)
Current therapy before bronchoscopy			
ICS (BDP pMDI)	6	3	3
LAMA end/or LABA or SAMA and/or SABA	15	13	9
Theophylline	3	5	3
Any medication	5	1	5
Therapy at discharge after bronchoscopy			
ICS (BDP pMDI)	11	5	9
LAMA end/or LABA or SAMA and/or SABA	16	13	13
Theophylline	3	5	4
Any medication	2	1	2

*ICS* inhaled corticosteroids, *BDP* beclomethasone dipropionate equivalent dose (μg/day), *pMDI* pressurized metered-dose inhaler, *LAMA* long-acting muscarinic antagonist, *LABA* long-acting beta2 agonist, *SAMA* short-acting muscarinic antagonist, *SABA* short-acting beta2 agonist. ICS use was excluded for a washout period of 1 month before bronchoscopy in all patients

### Immunohistochemistry in the bronchial epithelium

**Fig. 1:**
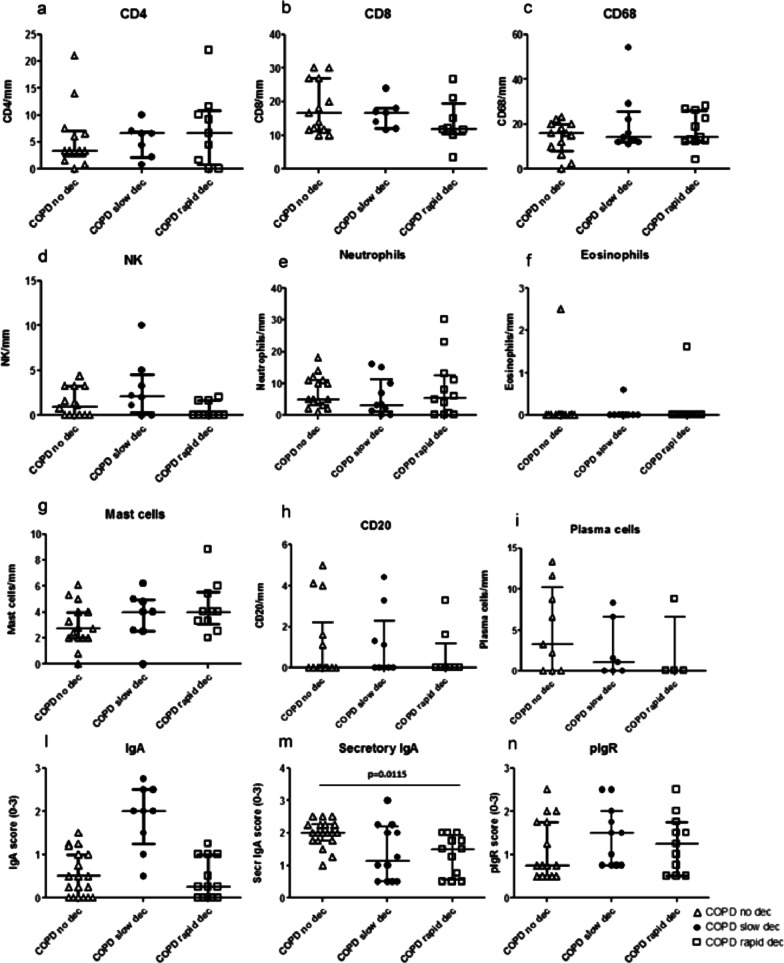
Quantitation of CD4 (**a**), CD8 (**b**), CD68 (**c**), natural killer cells (**d**), neutrophils (**e**), eosinophils (**f**), mast cells (**g**), B cells (CD20) (h), plasma cells (**i**), total IgA (**l**), secretory IgA (**m**) and polymeric immunoglobulin receptor (pIgR) (**n**) in the bronchial epithelium of non-decliners (n = 21), slow decliners (n = 14) and rapid decliners (n = 15) with COPD. Bar represents median value. Secretory IgA was significantly reduced in rapid decliners compared to non-decliners. Exact p value is reported in the graph (**m**)

**Fig. 2:**
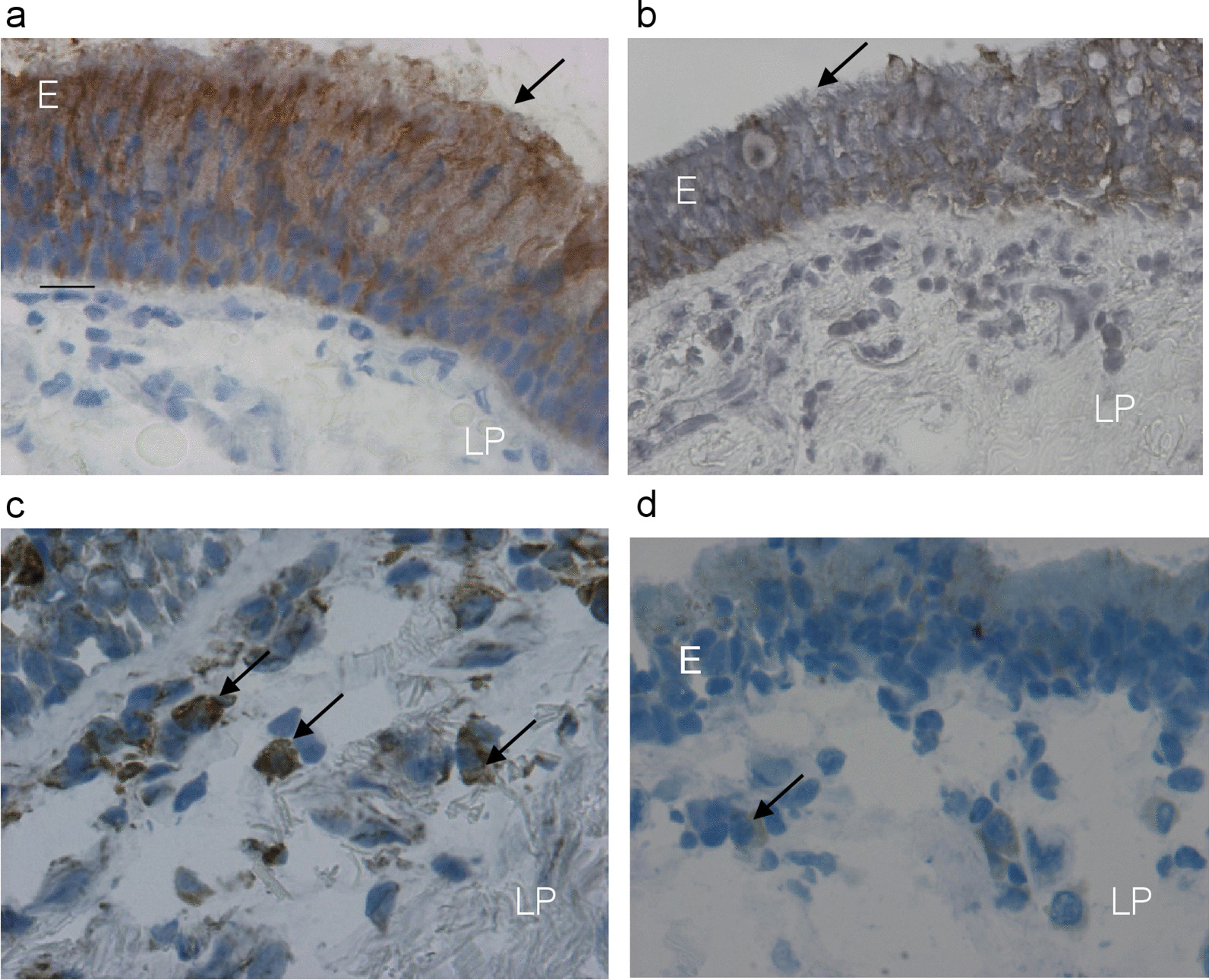
Photomicrographs showing bronchial mucosa from a representative non-decliner (**a**, **c**) and a rapid decliner (**b**, **d**) with COPD, immunostained for identification of secretory IgA protein (**a**, **b**) and plasma cells (**c**, **d**) in the epithelium (E) and bronchial lamina propria (LP). Results are representative of 21 non-decliners and 15 rapid decliners. Arrows (**a**, **b**) indicate immunopositivity in epithelial cells, which was reduced in rapid decliners (**b**), and immunostaining of plasma cells (**c**, **d**), which were reduced (**d**) in the lamina propria of rapid decliners with COPD. Bar = 20 micron

Numbers of inflammatory cells, CD4, CD8, CD68, Natural Killer (NK), neutrophils (elastase +), eosinophils (EG2 +), mast cells (tryptase +), CD20 (B cells), plasma cells infiltrating the bronchial epithelium (cells/mm) were similar in the three groups of COPD patients studied. Scored total IgA and pIgR, also, did not differ in the three groups (Fig. 1). Interestingly, the secretory IgA score was significantly reduced in rapid decliners compared to non-decliners (Kruskal–Wallis: p = 0.037, Mann–Whitney, p = 0.011) (Figs. 1m, 2a,b). The intra-observer (ADS) variability for secretory IgA score was 2.31%.

### Immunohistochemistry in the bronchial lamina propria

**Fig. 3:**
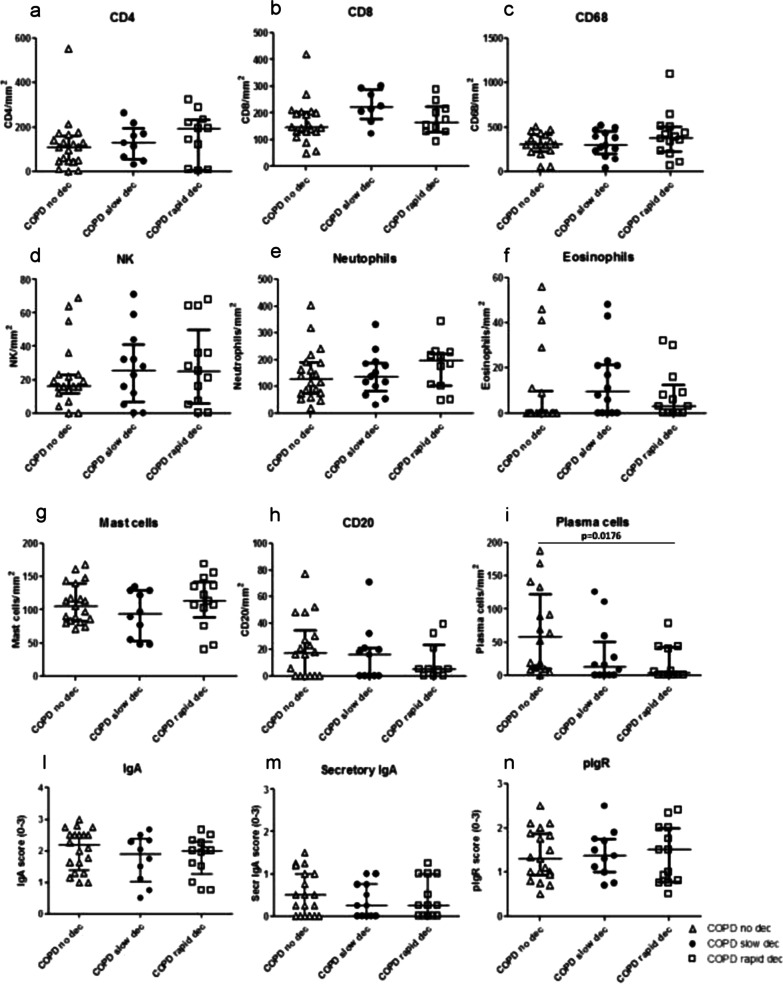
Quantitation of CD4 (**a**), CD8 (**b**), CD68 (**c**), natural killer cells (**d**), neutrophils (**e**), eosinophils (**f**), mast cells (**g**), B cells (CD20) (**h**), plasma cells (**i**), total IgA (**l**), secretory IgA (**m**) and polymeric immunoglobulin receptor (pIgR) (**n**) in the bronchial lamina propria of non-decliners (n = 21), slow decliners (n = 14) and rapid decliners (n = 15) with COPD. Plasma cell numbers were significantly reduced in rapid decliners compared to non-decliners. Exact p value is reported in the graph (**i**). Bar represents median value

In the bronchial lamina propria, we observed similar numbers of CD4, CD8, CD68, NK, neutrophils, eosinophils, mast cells, B cells (CD20), total and secretory IgA and pIgR (Fig. 3) across the three groups of patients. A modest and diffused pIgR immunopositivity in the lamina propria (LP) has also been observed. Since this receptor is mainly expressed in bronchial epithelial cells, its presence in LP may be due to epithelial leakage or damage and tissutal macrophage engulfment. Interestingly, numbers of plasma cells in the lamina propria were significantly decreased in rapid decliners compared to non-decliners (Kruskal–Wallis: p = 0.048, Mann–Whitney, p = 0.017) (Figs. 2c, d, 3i). The intra-observer (ADS) variability for plasma cells numbers in the lamina propria was 1.60%.

### Correlations between plasma cell count, total and secretory IgA and neutrophils in all COPD patients in bronchial biopsies

**Fig. 4:**
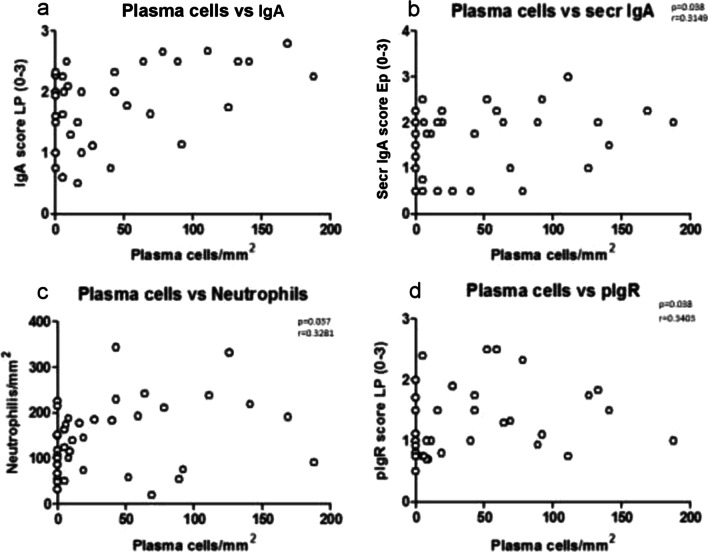
Regression analysis performed in all COPD patients (**a**–**d**) showing significant positive correlations. There is a significant positive correlation between the number of plasma cells/mm^2^ in the lamina propria and total IgA scored in the lamina propria (**a**) and secreted IgA scored in the epithelium (**b**). Plasma cells/mm2 in the lamina propria were also significantly correlated with number of neutrophils (**c**) and pIgR scored in the lamina propria (d). Correlation coefficients were calculated using the Spearman rank method

In all COPD patients, plasma cell numbers in the lamina propria correlated positively and significantly with total IgA score in lamina propria (Fig. 4a), secretory IgA score in epithelium (Fig. 4b) and pIgR score in lamina propria (Fig. 4d). Number of neutrophils in lamina propria correlated also positively and significantly with plasma cell numbers in the lamina propria (Fig. 4c). No other significant correlations were observed.

### ELISA tests for pIgR in the supernatant and cell lysate of LPS, H_2_O_2_ and IL-8 treated and non-treated 16HBE cells

**Fig. 5:**
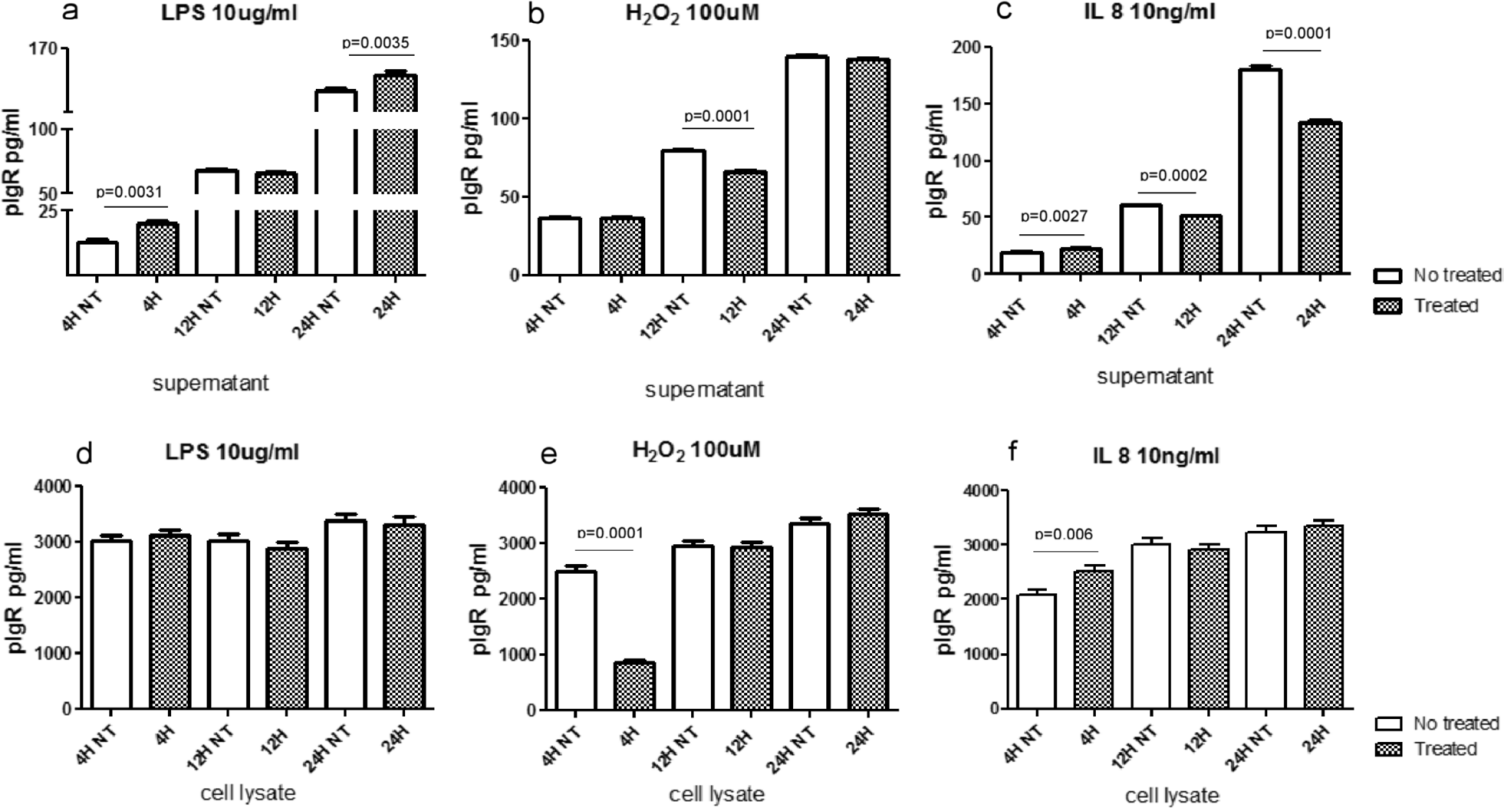
In vitro quantitation by ELISA tests of polymeric immunoglobulin receptor (pIgR) (secretory component) in the supernatants and cell lysate of normal primary human bronchial epithelial cells (16HBE) treated with lipopolysaccharide (LPS, 10 μg/ml), hydrogen peroxide (H_2_O_2_, 100 μM) and IL-8, 10 ng/ml). In the supernatant, LPS stimulation significantly increased pIgR protein release at 4 and 24 h after stimulation (**a**). IL-8 stimulation significantly increased pIgR protein release at 4 h, followed by a decrease at 12 and 24 h (**c**). Stimulation by H_2_O_2_ significantly decreased pIgR release at 12 h (**b**). In the cell lysate, LPS had no effect on pIgR expression at any time point or concentration studied (**d**). IL-8 stimulation significantly increased pIgR expression at 4 h after treatment (**f**). H_2_O_2_ significantly reduced pIgR concentrations at 4 h after treatment in the cell lysates of 16HBE cells (**e**)

Human bronchial epithelial cells (16HBE) were treated with LPS (10 µg/ml), H_2_O_2_ (100 µM) and IL-8 (10 ng/ml) for 4, 12, 24 h (Fig. 5). We analyzed the released form of pIgR in the supernatants and its intracellular component in cell lysates of stimulated and non-treated 16HBE cells.

In the supernatant, pIgR level (pg/ml) was enhanced by LPS at 4 and 24 h (Fig. 5a) and by IL-8 at 4 h after treatment, followed by a reduction at 12 and 24 h after IL-8 stimulation (Fig. 5c). H_2_O_2_ significantly reduced the level of pIgR at 12 h after stimulation in the supernatant of 16HBE cells (Fig. 5b).

In the cell lysate, LPS had no effect on pIgR expression at any time point or concentration studied (Fig. 5d). IL-8 stimulation significantly increased pIgR level (pg/ml) at 4 h after treatment (Fig. 5f). H_2_O_2_ significantly reduced pIgR concentrations at 4 h after treatment in the cell lysates of 16HBE (Fig. 5e).

## Discussion

We report here findings on the infiltration of inflammatory cells and expression of markers of humoral immune response in bronchial biopsies of patients with stable COPD with different degrees of lung functional decline. We evaluated three subgroups of COPD patients with rapid, slow or no lung functional decline monitored for an average of 5.8 years (all patients). The expression of secretory IgA was significantly reduced in bronchial epithelium and plasma cell numbers were significantly lower in the bronchial lamina propria of rapid decliners with stable COPD compared to non-decliners. No difference was found in the bronchial inflammatory cell infiltration due to CD4, CD8, CD68, CD20, NK, neutrophils, eosinophils, mast cells, polymeric immunoglobulin receptor (pIgR) in epithelium and lamina propria of rapid decliners compared to the other groups. Plasma cells in the lamina propria correlated positively with total IgA score in lamina propria of all patients. In vitro stimulation of 16HBE cells with LPS (10 μg/ml) and IL-8 (10 ng/ml) significantly increased pIgR expression in human bronchial epithelial cells while H_2_O_2_ (100 μM) significantly decreased it.

In the three subgroups (phenotypes) of COPD patients, follow-up duration for functional decline was similar (5.8 years) and ranged for all patients between 3 and 15 years. Similar FEV1% predicted values, RV%, DLCO/VA% and CT scored emphysema% were found, though the group with rapid functional decline showed a trend to lower DLCO/VA% and higher CT-scored emphysema%. DLCO% was slightly but significantly lower in rapid decliners compared to non-decliners (Table 1). It is likely that in a larger sample these lung functional parameters and CT-scored emphysema could would show statistically significant differences. However, this study was designed for different purposes, namely to analyze inflammatory cell infiltration and markers of humoral immune response, for which the sample size was sufficient, going by the literature [21, 22]. The subgroup of non-decliners improved their lung function over the 6.1 years (mean ± SD) study period. In an earlier study, 15% of patients assessed showed an improved lung function over the 3-year study period used for monitoring those patients [10], and the possibility that some patients might have improvements over time was also noted by Fletcher and Peto [9, 10] in historical studies on the natural history of COPD [9, 10]. Whether this is related to genetic differences or to response to treatment, numbers of exacerbations per year or smoking habit is not clear [10]. Interestingly, our rapid decliners showed a significantly higher pack/year consumption compared to slow and non-decliners (Table 1) while the percentage of frequent exacerbators in our rapid decliners (20%) was similar to the percentage amongst non-decliners (28%). These findings are in line with a previous study showing that current smokers had 21 ± 4 ml/year more decline compared to former smokers [10] while exacerbations during follow-up correlated to greater decline in FEV1 with a mean loss of 2 ± 0.5 ml/year per exacerbation [10], suggesting that smoking habit plays a major role in lung function decline.

In our study, the number of patients on the different therapies (mainly LAMA, LABA and ICS) before bronchoscopy and at discharge after bronchoscopy was similar in the three subgroups of COPD patients (Table 3), and after discharge therapy remained unvaried in the follow-up period in 80.9%, 78.6% and 100% of non-, slow, and rapid decliners, respectively (Table 3). Furthermore, ICS consumption at discharge tended to be higher in rapid decliners compared to the other groups (Kruskal–Wallis, p = 0.060). This seems in contrast with data from the literature, e.g. the TORCH study which indicated that decline in FEV1 may be reduced with regular treatment [7, 10]. Unfortunately our study cannot provide an answer to this point since it was designed with a limited number of patients to answer different questions. Our observation of a somewhat higher ICS consumption in rapid decliners at discharge was, in our opinion, mainly due to their severer outcome observed by clinicians who tended to increase the pharmacologic treatment for this subgroup of patients. We do not know whether a lower ICS treatment level than that observed in our rapid decliners would have even further aggravated the outcome. Based on the TORCH study [7, 10], we can surmise that it could be the case. Another aspect to consider is that the adherence to therapy of our patients was not precisely monitored prospectively, since our study was conducted retrospectively.

In our immunohistochemical analysis of bronchial biopsies from rapid, slow and non-decliners with COPD, we found no significant differences among groups concerning the bronchial inflammatory cell infiltration due to lymphocytes, NK cells, B cells, macrophages, mast cells, neutrophils and eosinophils, indicating that, at bronchial mucosal level, the increased systemic inflammation previously reported [11–13] is not evident. Interestingly, we observed decreased levels of secretory IgA in bronchial epithelium of rapid decliners compared to non-decliners (Fig. 1) and a decreased plasma cell count in the lamina propria of rapid decliners compared to non-decliners (Fig. 3). These data suggest that an impairment of the humoral immune response, inhibiting adherence of microorganisms to epithelium and their clearance, may be involved in the rapid lung function decline aggravating the disease state of these patients. In previous studies, a decreased level of secretory IgA or pIgR has been reported in association with severity of COPD. In severe COPD, a decreased pIgR bronchial expression correlated with airflow limitation and neutrophils [16, 17], while a reduced secretory component in both large and small airways correlated with the number of neutrophils in the glands in large airways, and with bronchial obstruction in small airways [16]. Areas of altered bronchial epithelium in COPD showed decreased secretory IgA and pIgR associated with increased inflammation [17]. Reduction of secretory IgA in small airways was associated with invasion of bacteria, NFkB activation, increased presence of macrophages and neutrophils and fibrotic remodeling of the small airways [23]. Specific IgA levels against *P. aeruginosa* were lower in stable non-colonized COPD compared to healthy subjects [24]. Another study found decreased pIgR in bronchial epithelium of severe COPD and decreased pIgR and IgA transcytosis in bronchial epithelium cell cultures of severe COPD vs. control subjects [25]. In our study, we did not observe a significant correlation between lung function decline level (ml/years) and post-β2 FEV1% predicted values (Spearman rank correlation, p = 0.249). In fact, the three groups of COPD patients had similar (mean ± SD) FEV1% predicted values notwithstanding their significantly different lung function decline (according to pre-defined cut-offs, see methods and Table 1). This suggests that, independently of the degree of severity of bronchial obstruction, the lower levels of secretory IgA in epithelium and plasma cells in lamina propria that we found in rapid decliners vs. non-decliners are a specific feature of functional decline and may be considered as markers of lung function decline in COPD patients.

In distal airways, IgA^+^ B cells numbers were increased in lymphoid follicles (LF) from severe COPD compared to control non-smokers [26] and the intra-LF IgA^+^ cells (%) were further increased in severe COPD compared to mild disease [26], presumably representing an adaptive immune response to microbial and/or self-antigens, particularly in severe disease [26]. However, in a different compartment of the lung, the IgA^+^ plasma cell numbers observed in bronchial mucosa of patients who died of COPD were lower compared to COPD patients who died from other causes [18]. More recently, plasma cells have been shown to be more numerous in the mucosal glands of patients with chronic bronchitis compared to asymptomatic smokers, but similar in number in the subepithelium [27]. In the bronchial mucosa (lamina propria) of our rapid decliners with COPD we observed a significant reduction of plasma cell numbers (Fig. 3) compared to non-decliners, which was also significantly and positively associated with the total IgA score in lamina propria and secretory IgA score in bronchial epithelium, confirming a relationship between the plasma cell numbers populating the bronchial mucosa and secreted IgA (Fig. 4). These findings coming from the large airways of COPD patients suggest an impairment of the humoral immune response developing not only in the more severe forms of COPD [9–12] or in patients who died from COPD [18] but also in the presence of a rapid lung function decline and deterioration of the disease state. Mechanisms of plasma cell maturation and differentiation need to be studied to further investigate the underlying molecular mechanisms [28–30] potentially involved in the reduction of plasma cells and secretory IgA in rapid decliners with COPD.

Our in vitro data, showing a decrease of polymeric Ig receptor both in supernatants and cell lysate samples after H_2_O_2_ treatment of 16HBE cells, a normal bronchial epithelial cell line, suggest speculatively, that oxidative stress caused by smoking habit, may play a role in reducing the IgA transcytosis from the normal bronchial mucosa to the airway lumen, starting in an early phase of bronchial obstruction.

In conclusion, we found an impairment of humoral immune response in COPD patients with rapid functional decline. This finding contributes to the phenotyping of COPD patients.

## Data Availability

The data from this study are available upon reasonable request.

## Abbreviations

COPD: Chronic obstructive pulmonary disease
FEV_1_: Forced expiratory volume in one second
FVC: Forced vital capacity
LP: Lamina propria
LPS: Lipopolysaccharides
pIgR: Polymeric immunoglobulin receptor
16HBE: Human bronchial epithelial cell line
DLCO: Diffusing capacity for carbon monoxide
DLCO/VA: Diffusing capacity for carbon monoxide/alveolar volume

## References

[1] Global Initiative for Chronic Obstructive Lung Disease (GOLD): global strategy for the diagnosis, management and prevention of chronic obstructive pulmonary disease. NHLBI/WHO workshop report. NIH Publication No 2701A. goldcopd.org.

[2] Wise RA (2006). The value of forced expiratory volume in 1 second decline in the assessment of chronic obstructive pulmonary disease progression. Am J Med.

[3] Anthonisen NR, Connett JE, Kiley JP, Altose MD, Bailey WC, Buist AS, Conway A, Enright PL, Kanner RE, O'Hara P (1994). Effects of smoking intervention and the use of an inhaled anticholinergic bronchodilator on the rate of decline of FEV1. The Lung Health Study. JAMA.

[4] Scanlon PD, Connett JE, Waller LA, Altose MD, Bailey WC, Buist AS (2000). Smoking cessation and lung function in mild-to moderate chronic obstructive pulmonary disease. The Lung Health Study. Am J Respir Crit Care Med.

[5] Donaldson GC, Seemungal TA, Bhowmik A, Wedzicha JA (2002). Relationship between exacerbation frequency and lung function decline in chronic obstructive pulmonary disease. Thorax.

[6] Decramer M, Celli B, Kesten S, Lystig T, Mehra S, Tashkin DP, UPLIFT investigators (2009). Effect of tiotropium on outcomes in patients with moderate chronic obstructive pulmonary disease (UPLIFT): a prespecified subgroup analysis of a randomised controlled trial. Lancet.

[7] Celli BR, Thomas NE, Anderson JA, Ferguson GT, Jenkins CR, Jones PW, Vestbo J, Knobil K, Yates JC, Calverley PM (2008). Effect of pharmacotherapy on rate of decline of lung function in chronic obstructive pulmonary disease: results from the TORCH study. Am J Respir Crit Care Med.

[8] Nishimura M, Makita H, Nagai K, Konno S, Nasuhara Y, Hasegawa M, Shimizu K, Betsuyaku T, Ito YM, Fuke S, Igarashi T, Akiyama Y, Ogura S (2012). Annual change in pulmonary function and clinical phenotype in chronic obstructive pulmonary disease. Am J Respir Crit Care Med.

[9] Fletcher CM, Peto R (1974). Letter: screening for diseases of the lung. Lancet.

[10] Vestbo J, Edwards LD, Scanlon PD, Yates JC, Agusti A, Bakke P, Calverley PM, Celli B, Coxson HO, Crim C, Lomas DA, MacNee W, Miller BE, Silverman EK, Tal-Singer R, Wouters E, Rennard SI (2011). Changes in forced expiratory volume in 1 second over time in COPD. ECLIPSE Investigators. N Engl J Med.

[11] Devanarayan V, Scholand MB, Hoidal J (2010). Identification of distinct plasma biomarker signatures in patients with rapid and slow declining forms of COPD. COPD.

[12] Higashimoto Y, Iwata T, Okada M, Satoh H, Fukuda K, Tohda Y (2009). Serum biomarkers as predictors of lung function decline in chronic obstructive pulmonary disease. Respir Med.

[13] Boutin M, Berthelette C, Gervais FG, Scholand MB, Hoidal J, Leppert MF, Bateman KP, Thibault P (2009). High-sensitivity nanoLC-MS/MS analysis of urinary desmosine and isodesmosine. Anal Chem.

[14] Mostov KE, Blobel GJ (1982). A transmembrane precursor of secretory component. The receptor for transcellular transport of polymeric immunoglobulins. Biol Chem.

[15] Pilette C, Ouadrhiri Y, Godding V, Vaerman JP, Sibille Y (2001). Lung mucosal immunity: immunoglobulin-a revisited. Eur Respir J.

[16] Pilette C, Godding V, Kiss R, Delos M, Verbeken E, Decaestecker C, De Paepe K, Vaerman JP, Decramer M, Sibille Y (2001). Reduced epithelial expression of secretory component in small airways correlates with airflow obstruction in chronic obstructive pulmonary disease. Am J Respir Crit Care Med.

[17] Polosukhin VV, Cates JM, Lawson WE, Zaynagetdinov R, Milstone AP, Massion PP, Ocak S, Ware LB, Lee JW, Bowler RP, Kononov AV, Randell SH, Blackwell TS (2011). Bronchial secretory immunoglobulin a deficiency correlates with airway inflammation and progression of chronic obstructive pulmonary disease. Am J Respir Crit Care Med.

[18] Soutar CA (1977). Distribution of plasma cells and other cells containing immunoglobulin in the respiratory tract in chronic bronchitis. Thorax.

[19] Di Stefano A, Caramori G, Barczyk A, Vicari C, Brun P, Zanini A, Cappello F, Garofano E, Padovani A, Contoli M, Casolari P, Durham AL, Chung KF, Barnes PJ, Papi A, Adcock I, Balbi B (2014). Innate immunity but not NLRP3 inflammasome activation correlates with severity of stable COPD. Thorax.

[20] Vestbo J, Hurd SS, Agustí AG, Jones PW, Vogelmeier C, Anzueto A, Barnes PJ, Fabbri LM, Martinez FJ, Nishimura M, Stockley RA, Sin DD, Rodriguez-Roisin R (2013). Global strategy for the diagnosis, management, and prevention of chronic obstructive pulmonary disease: GOLD executive summary. Am J Respir Crit Care Med.

[21] Sont JK, Willems LN, Evertse CE, Hooijer R, Sterk PJ, van Krieken JH (1997). Repeatability of measures of inflammatory cell number in bronchial biopsies in atopic asthma. Eur Respir J.

[22] Hattotuwa K, Gamble EA, O’Shaughnessy T, Jeffery PK, Barnes NC (2002). Safety of bronchoscopy, biopsy, and BAL in research patients with COPD. Chest.

[23] Polosukhin VV, Richmond BW, Du RH, Cates JM, Wu P, Nian H, Massion PP, Ware LB, Lee JW, Kononov AV, Lawson WE, Blackwell TS (2017). Secretory IgA deficiency in individual small airways is associated with persistent inflammation and remodeling. Am J Respir Crit Care Med.

[24] Millares L, Martí S, Ardanuy C, Liñares J, Santos S, Dorca J, García-Nuñez M, Quero S, Monsó E (2017). Specific IgA against *Pseudomonas aeruginosa* in severe COPD. Int J Chron Obstruct Pulmon Dis.

[25] Gohy ST, Detry BR, Lecocq M, Bouzin C, Weynand BA, Amatngalim GD, Sibille YM, Pilette C (2014). Polymeric immunoglobulin receptor down-regulation in chronic obstructive pulmonary disease. Persistence in the cultured epithelium and role of transforming growth factor-β. Am J Respir Crit Care Med.

[26] Ladjemi MZ, Martin C, Lecocq M, Detry B, Nana FA, Moulin C, Weynand B, Fregimilicka C, Bouzin C, Thurion P, Carlier F, Serré J, Gayan-Ramirez G, Delos M, Ocak S, Burgel PR, Pilette C (2019). Increased IgA expression in lung lymphoid follicles in severe chronic obstructive pulmonary disease. Am J Respir Crit Care Med.

[27] Zhu J, Qiu Y, Valobra M, Qiu S, Majumdar S, Matin D, De Rose V, Jeffery PK (2007). Plasma cells and IL-4 in chronic bronchitis and chronic obstructive pulmonary disease. Am J Respir Crit Care Med.

[28] Curtis JL (2017). A hairline crack in the levee: focal secretory IgA deficiency as a first step toward emphysema. Am J Respir Crit Care Med.

[29] Nguyen DC, Joyner CJ, Sanz I, Lee FE (2019). Factors affecting early antibody secreting cell maturation into long-lived plasma cells. Front Immunol.

[30] Khodadadi L, Cheng Q, Radbruch A, Hiepe F (2019). The maintenance of memory plasma cells. Front Immunol.

